# Psychological barriers moderate the attitude-behavior gap for climate change

**DOI:** 10.1371/journal.pone.0287404

**Published:** 2023-07-05

**Authors:** João Vieira, São Luís Castro, Alessandra S. Souza

**Affiliations:** Center for Psychology, Faculty of Psychology and Education Sciences, University of Porto, Porto, Portugal; University of Castilla-La Mancha: Universidad de Castilla-La Mancha, SPAIN

## Abstract

Behavioral change has been increasingly recognized as a means for combating climate change. However, being concerned about climate problems and knowing the importance of individual actions in mitigating them is not enough for greater adherence to a more sustainable lifestyle. Psychological barriers such as (1) finding change unnecessary; (2) conflicting goals; (3) interpersonal relationships; (4) lack of knowledge; and (5) tokenism have been proposed as an explanation for the gap between environmental attitudes and actions. Yet, so far, this hypothesis has remained untested. This study aimed to assess if psychological barriers moderate the association between environmental attitudes and climate action. A sample of Portuguese individuals (N = 937) responded to a survey measuring climate change beliefs and environmental concerns as an index of environmental attitudes, a scale of self-reported frequency of environmental action, and finally, the dragons of inaction psychological barrier scale. Our participants revealed generally elevated positive environmental attitudes. These attitudes were positively and moderately related to greater self-reported frequency of environmental action in areas such as reusing materials, reduced consumption of animal products, water and energy saving, and airplane use, but not driving less. Critically, the association between attitudes and behavior was negatively moderated by psychological barriers for the reuse, food, and saving domains, but not for driving or flying. In conclusion, our results corroborate the assumption that psychological barriers can partly explain the attitude-behavior gap in the climate action domain.

## Introduction

Global surface temperatures are rising at unprecedented levels due to an increase in the concentration of greenhouse gases produced by human activity [1, 2]. This warming trend has propelled an increased frequency of extreme weather events, which has and will continue to have a significant impact on human physical [3, 4] and mental health [5, 6]. Despite the irreversibility of some of the consequences of climate change, drastically reducing anthropogenic greenhouse gas emissions is imperative to prevent global warming from reaching the 1.5°C mark [7]. Reducing these emissions will require major changes in human behavior toward a more sustainable lifestyle [8–11]. The frequency with which people engage in pro-environmental behaviors is assumed to be related to their environmental attitudes [12–15], yet the link between environmental attitudes and behavior has been proposed to be weakened by several psychological barriers [16–19]. To the best of our knowledge, no previous studies directly evaluated whether psychological barriers moderate the association between attitudes and pro-environmental behavior. The goal of the present study is to fill this gap by measuring environmental attitudes, self-reported frequency of environmental behaviors, and psychological barriers to assess whether psychological barriers partly explain the relationship between attitudes and behavior.

### Environmental behaviors and attitudes

Household consumption is a country’s leading contributor to greenhouse gas emissions (ca. 65% to 75% of the emissions), especially in the high-income countries of Europe and North America [10, 11]. Household carbon emissions are defined as the intensity of carbon emissions resulting from individuals’ and groups’ behavioral activities that support their lifestyles [9, 20]. In Western countries, the main contributors to these emissions are transportation, housing, and food consumption [9–11, 21]. Therefore, changes in demand and consumption patterns of households have tremendous climate change mitigative potential [8, 11]. Shifting transportation and dietary habits, as well as encouraging energy-saving at home, is a fundamental challenge requiring a deep understanding of the psychological factors involved in lifestyle choices, attitudes toward the environment, and opportunity costs for ecological behaviors [22, 23]. In the present study, we measured the self-reported behavior frequency in the most relevant domains of ecological behavior, namely, transportation, food choices, and household energy and water consumption to serve as a first step toward better understanding ecological behavior.

Psychological models have been put forth to explain how psychosocial variables function together to stimulate the motivation to act [12, 15]. Most of these models agree that positive attitudes towards the environment, which are related to feelings of personal moral responsibility to act in favor of the environment (i.e., environmental personal norms), are necessary for pro-environmental behavior. The widely used value-belief-norm theory [24, 25] and the more recent comprehensive action-determination model [12, 26] postulate that our personal norms are activated when we feel that something valuable is being threatened. Hence, activating each individual’s personal norms related to the environment requires the interaction between environmental beliefs and values.

To believe in environmental problems, an individual needs to possess enough knowledge to recognize the existence of environmental issues [27–29]. Beliefs can be about recognizing the presence of an unnatural climate change, knowing its negative impacts, and the human responsibility for it. Additionally, it also includes believing in personal responsibility. Beliefs are sometimes directly associated with pro-environmental behavior [22, 30], but they mainly impact intention and willingness through interaction with other constructs such as environmental values, social norms, and knowledge [27, 31, 32].

Values guide individuals through the selection and evaluation of behaviors [33] and dictate what individuals are concerned about when making decisions [34, 35]. In the environmental context, egoistic, altruistic, and biospheric concerns are particularly relevant [35–38]. While egoistic concerns guide individuals toward self-enhancement, altruistic concerns are associated with the benefit of others, and biospheric concerns fall on valorizing nature and the environment. Biospheric concerns appear to have the strongest association with pro-environmental action, followed by altruistic and, finally, egoistic ones [22, 32, 35, 38–40], but all three types of concerns are usually correlated [35, 41–43]. Thus, when an individual expresses concern for environmental problems, their worries are oriented to the following three possible valued objects: the self, other people, and all living beings. Believing that something valued is being threatened is an important aspect of developing favorable environmental attitudes, even when those concerns are at a personal level (egoistic) [41]. The present study assessed environmental attitudes by measuring climate change-related beliefs and environmental concerns. We predicted that believing in the negative impact of climate change paired with displaying environmental concerns for either the self, others, or nature should lead to favorable attitudes regarding climate change.

### The attitude-behavior gap

Studies often report a moderate relationship between attitudes and pro-environmental behaviors [e.g., r = 0.36 in 12, r = 0.482 in 22]. Despite this relationship, a growing number of studies reports that being concerned or feeling responsible for acting in favor of the environment is not enough to increase pro-environmental behavior [12, 14, 18, 44–47]. This gap has been named the *attitude-behavior gap* or the *value-action gap*. Terms such as *belief-action gap*, *knowledge-action gap*, and *attitude-behavior inconsistency* have also been employed [18]. Critically, not all pro-environmental actions are similarly affected by attitudes [14]. The higher the perceived costs of an action, the lesser the effect attitudes have on actual behaviors [48]. This could be explained by environmentally concerned individuals feeling dissatisfied when their actions do not correspond to their values and attitudes, yet they also do not want to bear significant personal costs or discomfort in pursuing them. Accordingly, the low-cost hypothesis states that the lower the personal costs in a situation, the easier it is for actors to transform their attitudes into corresponding behavior [48, 49]. The cost of an action usually depends on the efforts necessary to execute it; for example, recycling can increase in cost if no nearby structures allow for the correct waste disposal and people would need to walk or drive more to dispose of their waste [50]. Decreasing meat consumption [51–53] and reducing personal vehicle use [54–56], two of the behaviors with the highest carbon emissions, are usually perceived as highly costly behaviors because sustainable alternatives are seen as less convenient (e.g., unappealing vegetarian meal alternatives or uncomfortable public transportation).

The weak association between attitudes and behavior could be partially explained by measurement inadequacies such as social desirability biases, temporal gaps, and faulty instruments [18]. The literature has also increasingly pointed to the relevance of assessing structural barriers (i.e., external systemic or infrastructure barriers) such as the accessibility and condition of sustainable alternatives, which can also hinder action-taking by aware and concerned individuals. For example, in the case of household carbon emissions, the cost of more sustainable items like organic and vegan food [57, 58], a perceived lack of available time [45, 59, 60], and difficulty in accessing structures or products in some areas [61, 62] have been pointed as barriers preventing concerned individuals from acting in accordance with their attitudes. However, even in situations where contextual and external factors do not heavily constrain individuals, the frequency of environmental action may still be low [14, 16].

### Psychological barriers

Psychological barriers have been put forth to explain why concerned individuals do not act according to their attitudes even when not restricted by structural barriers [16]. For example, a mixed method analysis by Lorenzoni et al. [63] showed that, at the individual level, uncertainty, distrust in information sources, externalizing responsibility, conflicting priorities, and fatalism were used as justifications for individual inaction in the climate context. Gifford [16] reviewed *The Dragons of Inaction*, an enumeration and explanation of 29 different psychological factors that impede adherence to climate change mitigation behaviors. These psychological barriers were categorized into seven groups: Limited Cognition, Ideologies, Comparison with others, Sunk costs, Discredence, Perceived Risks, and Limited Behavior.

Gifford and Chen [17] evaluated the impact of some of these psychological barriers on intentions to engage in mitigative food choices (e.g., purchase of organically grown food). They observed that the dismissal of anthropogenic climate change evidence was the strongest perceived barrier to mitigation, the second being incompatible financial and time investments, followed by satisfaction with current mitigation behaviors. Gifford’s psychological barriers were also negatively associated with self-reported energy conservation: the existence of other conflicting goals and aspirations had the strongest negative correlation with energy conservation behaviors, and interpersonal influences the weakest [64].

Each of these studies pointed to a different structure of psychological barriers. More recently, Lacroix et al. [19] developed a short scale to measure psychological barriers to pro-environmental behavior that could be applied across multiple domains of environmental behavior, the *Dragons of Inaction Psychological Barriers* (DIPB) scale. After a set of three studies, the final scale measured five barriers: (a) *Change Unnecessary*, that is, the denial of environmental problems and of the necessity to act; (b) *Conflicting Goals and Aspirations*, comprising limited time, sunk costs, and difficulty in changing habits; (c) *Interpersonal Relationships*, covering the fear of social disapproval or criticism; (d) *Lacking Knowledge*, representing not knowing how to change; and (e) *Tokenism*, the belief that no more personal investment is needed to change. The five barriers model was invariant for a wide range of behaviors that the authors considered as high-difficulty (e.g., eating less meat and driving less) and low-difficulty (e.g., recycling, saving water, saving energy, buying green); nonetheless, the impact of each barrier varied across behaviors. The interpersonal relationships barrier was the strongest for eating less meat, the conflicting goals and aspirations barrier was more substantial for driving less, and the lacking knowledge barrier was the strongest for buying green. Tokenism did not differ across behaviors. The DIPB scale was also adapted to the Colombian population, with a similar five-factor structure adequately describing the data [65]. Wang et al. [66] showed that the DIPB was associated with inaction and mediated the relation between positive emotions towards nature (awe) and climate change inaction. Here we adapted the DIPB scale for the Portuguese population to measure the five psychological barriers identified in these previous studies.

### The current study

Psychological barriers have been pointed out as a possible explanation of inaction in individuals concerned about climate change but not effectively acting on their beliefs [16]. The DIPB scale was created to measure the effect of psychological barriers on concerned individuals under the assumption that they may help explain the attitude-behavior gap [19]. However, to the best of our knowledge, no study has assessed whether psychological barriers help explain this gap.

**Fig 1:**
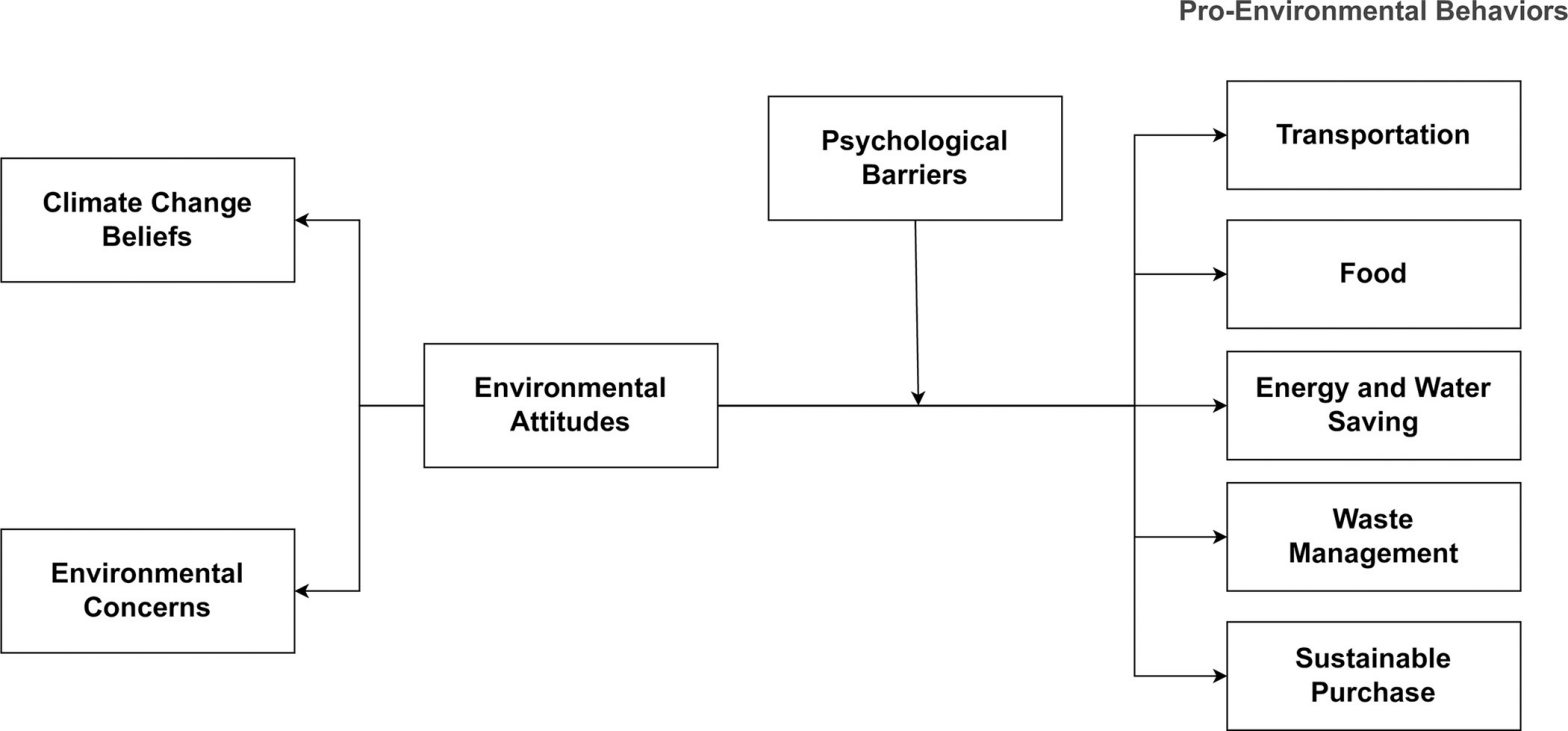
The model framework assessed in this study.

The current study aimed to test whether psychological barriers, measured by the DIPB scale, partially explain the gap between environmental attitudes and pro-environmental behaviors. Our goal was to test the model illustrated in Fig 1. Attitudes were measured by climate change beliefs and environmental values [12]. To include a wide range of pro-climatic behavior from more to less impactful, we measured the self-reported frequency of pro-environmental behaviors in the following domains: transportation, food, energy and water saving, waste management, and sustainable purchase. We expected to find that behaviors related to transportation and food would be less affected by attitudes and would be hindered more by psychological barriers than the lower-investment counterparts (waste management, purchase, and conservation).

We collected data in Portugal. Previous surveys have shown that the Portuguese are aware and concerned about climate change and the environment [30, 67, 68]. Nevertheless, the country has a low carbon-emission reduction performance [69], and the citizens report engaging mainly in low-impact behaviors [67]. As a member of the Organization for Economic Cooperation and Development (OECD) and the European Union, Portugal shares characteristics with similar countries [10], where citizens living in urban contexts have higher levels of education, more accessible public transportation systems and a better supply of low carbon products. These characteristics are usually associated with a reduction of structural barriers for environmental action, thus making Portugal a suitable context to study the attitude-behavior gap and the role of psychological barriers.

## Materials and methods

### Participants

The study was distributed online between November 2021 and March 2022. We recruited 937 Portuguese-speaking individuals residing in Portugal via the University of Porto’s mailing list, various online groups, and the promotion ad feature of Facebook and Instagram. Overall, 650 participants identified as women (69%), 280 as men (30%), and seven as other/no response (1%). Ages ranged from 18 to 80, with the average being 36 years (*SD* = 16). Most participants reported having some form of higher education degree (67%), 290 concluded high school (31%), and only 18 did not complete secondary education (2%). More than half (56.7%) resided in Portugal’s two most populated and urbanized cities (Porto and Lisbon). Participants completed a written online informed consent form at the beginning of the study (i.e., they read and clicked on consent statements before moving on to the main study) and were debriefed at the end. If participants did not consent to the terms of participation, the study was ended before completion of the questionnaire. The Faculty of Psychology and Education Sciences ethics committee approved the research protocol (Ref.ª 2021/09-08).

### Instruments

#### Environmental concerns

We used the *Environmental Concern Items* from Schultz [35], which has been referred to as a robust measurement of environmental concern [70]. It comprises 12 items, divided into four altruistic (e.g., "All people"), four egoistic (e.g., "My lifestyle”), and four biospheric (e.g., "Animals") domains of environmental concern. Participants rated the 12 items using a scale from 1 (not important) to 7 (supreme importance) in response to the question: *"I am concerned about the environmental problems because of the consequences for…*.*"*. The instrument has good reliability and correlates with other measures of environmental attitude, self-reported pro-environmental behavior, and social value orientation [35, 41, 70]. Items are shown in Table 1.

**Table 1:** Items, descriptive statistics, and ordinal alpha for scales used to measure environmental concerns and climate change beliefs.

Scale	Factors and Items	*M*	*SD*	α if dropped
Schultz (2001)	**Egoistic Concerns** (α = **.91)**			
	1. My lifestyle	5.06	1.68	.91
	4. Me	5.49	1.53	.85
	7. My health	6.05	1.27	.88
	10. My future	5.98	1.31	.87
	**Altruistic Concerns (**α **= .91)**			
	2. People in my country	5.79	1.33	.90
	5. All people	6.32	1.05	.87
	8. Children	6.39	1.08	.88
	11. Future generations	6.62	0.94	.87
	**Biospheric Concerns (**α **= .97)**			
	3. Plants	6.12	1.23	.97
	6. Animals	6.43	1.02	.95
	9. Marine life	6.46	1.02	.96
	12. Birds	6.33	1.13	.95
Christensen & Knezek (2015)	**Beliefs (α = .94)**			
	1. I believe our climate is changing (believe)	4.81	0.53	.93
	2. I am concerned about global climate change (concern)	4.77	0.60	.93
	3. I believe there is evidence of global climate change (evidence)	4.76	0.59	.93
	4. Global climate change will impact our environment in the next 10 years (impact)	4.73	0.66	.93
	5. Global climate change will impact future generations (future)	4.89	0.47	.92
	6. The actions of individuals can make a positive difference in global climate change (difference)	4.31	0.88	.94
	7. Human activities cause global climate change (humans)	4.77	0.59	.93
	8. Climate change has a negative effect on our lives (negative)	4.72	0.65	.93
	9. I can do my part to make the world a better place for future generations (my part)	4.63	0.67	.94

*Note*. Terms presented in parentheses were used to identify the items in the model visualizations.

#### Climate change beliefs

We measured climate change beliefs by adapting items from *The Climate Change Attitude Survey scale* [71]. The belief sub-scale comprises nine items rated on a Likert scale ranging from 1 (strongly disagree) to 5 (strongly agree). The used items are presented in Table 1.

#### Frequency of pro-environmental behaviors

We built a questionnaire to measure the self-reported frequency of the most relevant pro-environmental behaviors in the following domains: transportation, food, energy and water saving, waste management, and sustainable purchase [11, 67, 72, 73]. The set of 21 items (see Table 2) was rated regarding their frequency on a Likert scale from 1 (rarely) to 5 (very often). We asked participants to rate the frequency of each action at the moment of the scale completion (during the COVID-19 pandemic) and what they believe was the frequency of their behavior before the onset of the COVID-19 pandemic. We intended to assess if behaviors changed because of the ongoing pandemic situation (which could particularly affect transportation behaviors). Responses were similar for both questions; hence, we will only present the results regarding the current self-reported frequency of action.

**Table 2:** Items and descriptive statistics for the scale used to measure the frequency of pro-environmental behaviors.

Items	*M*	*SD*
**Transportation**		
1. I use public transportation	3.01	1.30
7. I walk or cycle instead of driving	3.06	1.24
13. I avoid long-distance flights (lasting six hours or more)	3.87	1.41
18. I prefer to travel by train/bus/boat instead of plane	3.45	1.29
**Food**		
4. I reduce the consumption of meat (e.g., beef, pork, poultry)	3.42	1.22
10. I reduce the consumption of eggs and dairy products	2.83	1.27
16. I reduce the consumption of fish	2.95	1.28
20. I increase the consumption of vegetables and fruits.	4.12	0.91
**Energy Saving**		
2. I turn off the lights when leaving the room	4.72	0.55
8. I cut down on heating or air conditioning to limit energy use ^a^	4.02	1.02
14. I turn the TV off when leaving the room	4.48	0.83
19. I try to reduce our domestic energy consumption (insulated home, installed solar panels, switched to LED lights) ^a^	3.79	1.13
**Water Saving**		
3. I limit my time in the shower in order to conserve water ^a^	3.94	1.02
9. I wait until I have a full load to use the washing machine or dishwasher ^a^	4.74	0.58
15. I close the tap while brushing my teeth	4.66	0.74
**Waste Management**		
5. I try to reduce the production of waste	3.95	0.98
11. I separate the various types of garbage for recycling	4.48	0.95
17. I cut down on my consumption of disposable items (e.g., plastic bags, disposable masks)	3.80	0.92
21. I reuse food leftovers ^a^	4.57	0.72
**Sustainable Purchase**		
6. I buy products that are not tested on animals ^a^	3.46	1.10
12. I buy local products	3.57	0.79

*Note*. ^a^ These items were removed from the final models.

#### Psychological barriers

We used the 22-item DIPB scale [19] and two additional items that were removed from the original final scale (items 23 and 24) but were recommended to be assessed in contexts where the government plays an important role. We included these items to assess if they could bring relevant information to measuring psychological barriers in Portugal since the Portuguese tend to attribute to local and national government a high (larger than the European Union average) responsibility in fighting climate change [74]. Each item was answered on a 7-point Likert scale, ranging from 1 (strongly disagree) to 7 (strongly agree). All items are presented in Table 3. The DIPB scale assesses five psychological barriers: (a) Change Unnecessary, (b) Conflicting Goals and Aspirations, (c) Interpersonal Relationships, (d) Lacking knowledge, and (e) Tokenism.

**Table 3:** Items, descriptive statistics, and ordinal alpha for the psychological barriers scale.

Order	Item	*M*	*SD*	α if dropped
	**Change Unnecessary (α = .91)**			
1.	1. There’s not much point in me making this change because I feel confident that technological innovators will solve environmental problems.	1.90	1.25	.93
6.	2. Humans are powerless when it comes to saving the earth, so there is no need to change	1.45	1.07	.90
11.	3. These problems are so far in the future, so there is no need to act.	1.24	0.72	.87
16.	4. Changes like this are not really necessary for me because environmental conditions are likely to remain ok in my area.	1.38	0.86	.88
21.	5. There is no pressing need to change because nature has great resiliency, and our actions are trivial.	1.37	0.96	.88
	**Conflicting Goals and Aspirations (α = .84)**			
2.	6. Making this change would interfere too much with my other goals in life.	2.56	1.64	.80
7.	7. I’m concerned that this change will take up too much of my time	2.77	1.91	.84
12.	8. I can’t change because I’m invested in my current lifestyle.	2.40	1.68	.80
17.	9. These issues are important to me but it’s too hard to change my habits.	3.11	1.91	.82
22.	10. I haven’t changed because I’m afraid this wouldn’t work.	2.17	1.65	.79
	**Interpersonal Relations (α = .88)**			
3.	11. Making this change would be criticized by those around me.	2.08	1.61	.87
8.	12. I would be letting certain people down if I made this change.	1.55	1.14	.82
13.	13. I’m worried that my friends would disapprove if I made this change.	1.40	1.08	.86
18.	14. If I made the necessary change, I would probably be embarrassed when others noticed what I was doing	1.30	0.83	.84
	**Lacking Knowledge (α = .70)**			
4.	15. There’s so much information out there that I am confused about how to make this change.	2.02	1.53	.57
9.	16. I don’t understand enough of the details about how to make this change.	2.27	1.70	.47
14.	17. I’d like to change but I’m not sure where to begin.	2.61	1.80	.52
19.	24. It’s the government’s responsibility to regulate this change. ^a^	4.76	2.00	.85
	**Tokenism (α = .80)**			
5.	18. The pro-environmental efforts that I currently engage in make further changes unnecessary.	1.93	1.37	.75
10.	19. I’ve already made sacrifices to solve environmental problems, so there is no need for me to do more.	1.48	0.97	.72
15.	20. I previously have made important effort in this, so there is no need for me to make further changes	1.40	0.88	.72
20.	21. My environmental actions already make enough of a difference ^a^	3.31	1.75	.80
23.	22. It’s not fair for me to change when really, it’s industry that’s causing the majority of environmental problems.	2.57	1.79	.77
24.	23. The government should make it easier for me to change, if it really has the best interest of the environment in mind. ^a^	3.92	2.13	.82

*Note*. ^a^ These items were removed from the final models. Items 23 and 24 were not part of the original final DIPB scale.

Before answering the scale, participants first selected one behavior from a list of five pro-environmental behaviors to serve as a reference while rating each item. The selected behavior should be one that participants believed was necessary to help the environment but which they were not currently doing or were not doing often enough. The behaviors were: (a) eating less meat; (b) taking public transport, cycling, or walking rather than driving; (c) reducing water use (e.g., taking shorter showers, repairing leaks); (d) making more eco-friendly purchases, and (e) recycling all items permitted by their local programs. Due to a programming error, one domain of environmental behavior formerly tested in the DIPB scale (i.e., wearing a sweater rather than turning up the heat in the winter) was not included in the list.

### Procedure

We requested permission from the original authors of the scales to use and adapt them in the present study. Our first step was to translate all items into Portuguese. We focused our translation on capturing relevant concepts to measure our research questions [75, 76]. All three authors translated all scales independently and then discussed the three translations to settle on a final version of each item. Afterward, an environmental science major assessed the translation and deemed the terminology suitable. The translated items used in the present study are available on our OSF page: https://osf.io/j9th2/

Completion of the questionnaire took, on average, 15-min. Participants completed the scales in the following order: (1^st^) environmental concern, (2^nd^) climate change beliefs, (3^rd^) frequency of ecological behaviors, (4^th^) the psychological barriers scale, and (5^th^) demographics questions.

### Data analysis

We performed confirmatory factor analysis to evaluate the structure of the responses to the environmental concerns, climate change beliefs, and psychological barriers scales. For the frequency of pro-environmental behavior, we first ran an exploratory factor analysis to determine the structure of the measurement of these behaviors, followed by a confirmatory factor analysis with the final identified model. All analyses were performed using R. 4.1.3 [77]. We used the *lavaan* package [78] for the confirmatory factor analysis, and the package *psych* [79] for the exploratory factor analyses and the reliability of our measurements. Finally, we used the base *R Stats* package to compute the moderation analysis using the *lm* function [77].

We first tested the multivariate normality of our data using the Energy test and Mardia’s multivariate skewness and kurtosis tests. Given that our data was ordinal, non-normal, and the sample size was large, we employed a diagonally weighted least squares (DWLS) estimation based on a polychoric correlation matrix. This method produces more accurate factor loadings estimates than the more commonly used maximum likelihood estimation method [80, 81].

To assess model adequacy, we used the following model fit indices: chi-square goodness-of-fit statistic (χ2), the root-mean-square error of approximation (RMSEA), Bentler’s comparative fit index (CFI), and the standardized root-mean-square residual (SRMR). For χ2, a small, non-significant value indicates a good fit. However, χ2 is known to be affected by sample size. RSMEA smaller than 0.06 indicates a good fit, and of 0.08 or less, a reasonable fit. For the CFI, values higher than 0.95 indicate a good fit, and values between 0.90 and 0.95 indicate an adequate fit. Finally, for the SRMR, a value below 0.08 indicates an acceptable fit. The cutoff criteria were based on Hu and Bertler [82]. Note that these cutoff criteria are not static and should be weighted considering the data since the index values can be influenced by sample size, the number of variables analyzed, and missing data [83].

Finally, we calculated the reliability of each of our measurements. Since our data is ordinal and non-normal, we report ordinal alpha (α), ordinal omega total (ωt), and Guttman’s greatest lower bound (GLB) [84].

All the materials, data, and analysis scripts used are publicly available at: https://osf.io/j9th2/

## Results

Table 1 presents descriptive statistics and reliability of the Environmental Concerns and Climate Change Beliefs items. Table 2 shows these values for the Frequency of Pro-Environmental Behavior, and Table 3 for the Psychological Barrier scales. The item numbering represents the order in which items were presented in the scale for rating. For the Psychological Barrier scale, we present the item order used in this study and the scale’s original numbering. We described each item’s mean (M) and standard deviation (SD). Ordinal alpha (α) for each initial factor (before item removal) is presented after the factor’s name and a column with the alpha value after removing different items inside that factor. For the factors with only two items, we presented the item’s correlation.

### Environmental attitudes

Our goal was to create an environmental attitude second-order latent factor consisting of a factor of environmental concerns and a factor of climate change beliefs. We first tested each of these constructs independently, with one model for responses in the environmental concerns scale, and another for the climate change beliefs scale. Regarding environmental concerns, participants reported higher levels of biospheric concerns (*M* = 6.33, *SD* = 1.52), followed by altruistic (*M* = 6.26, *SD* = 1.11), and lastly egoistic concerns (*M* = 5.56, *SD* = 1.15), replicating prior results [35]. CFA revealed that the 3-factor solution proposed by Schultz [35] had an adequate fit, χ2 (51) = 90.119, CFI = 0.991, RMSEA = 0.029 [0.019, 0.038], SRMR = 0.060. Concerning the Climate Change Belief scale, overall, participants reported high levels of agreement with the items (*M* = 4.71, *SD* = 0.64), indicating that they believed in climate change. Following prior research [71], we tested whether these items formed a single factor, yet this model had a SRMR value above the cut-off, χ2 (27) = 60.429, CFI = 0.968, RMSEA = 0.036 [0.024, 0.049], SRMR = 0.081. The inclusion of a residual covariation between items *6*. *The actions of individuals can make a positive difference in global climate change* and *9*. *I can do my part to make the world a better place for future generations*, was necessary to obtain an acceptable and very good fit value, χ^2^ (26) = 11.744, CFI = 1, RMSEA = 0.00 [0.00, 0.00], SRMR = 0.042. These items are highly correlated (b = 0.58) and *they* are the only ones covering the belief in individual contributions to fighting climate change. This change aligns with previous proposals dividing beliefs into the dimensions of awareness of consequences and ascription of responsibility [12]. Reliability results for our final factors were as follows: Egoistic Concerns (α = .91, ωt = .93, GLB = .91); Altruistic Concerns (α = .91, ωt = .94, GLB = .95); Biospheric Concerns (α = .97, ωt = .91, GLB = .98); Beliefs (α = .94, ωt = .97, GLB = .98).

**Fig 2:**
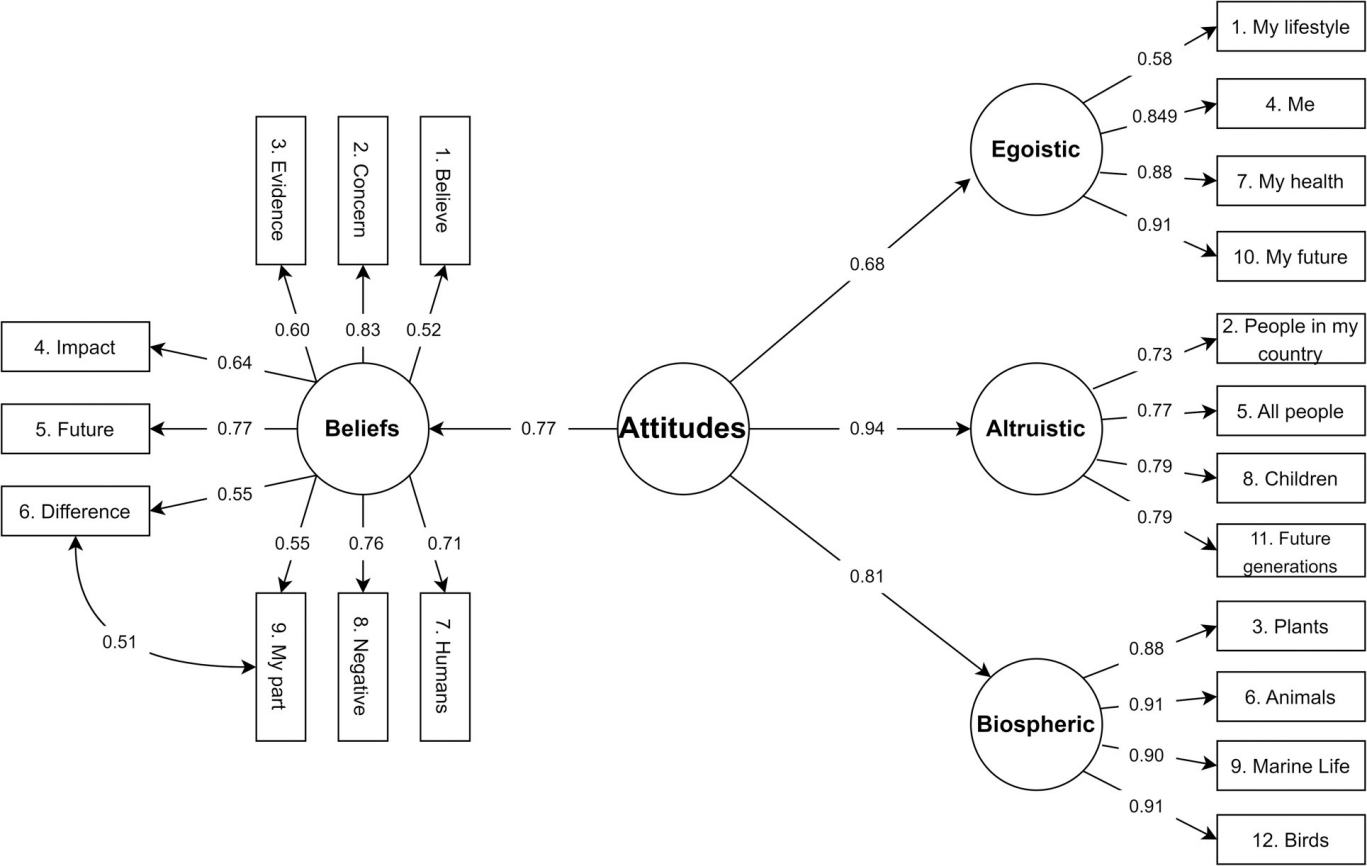
Environmental attitude model. *Note*. Loadings and factor correlations are given as standardized parameters.

Next, we created a second-order latent variable of Attitudes on which the three factors of environmental concerns (Egoistic, Altruistic, Biospheric) and the Beliefs factor loaded. The model had good fit: χ^2^ (184) = 226.295, CFI = 0.995, RMSEA = 0.016 [0.007, 0.022], SRMR = 0.064. Despite some research pointing at the weaker relationship between egoistic concerns and environmental-related attitudes and behaviors [70], we retained this factor in our model since it positively correlated with the other concern factors and loaded on the second-order latent variable of Attitudes [35, 41]. Fig 2 presents the full model and the standardized loadings.

### Environmental behaviors

We assessed the self-reported frequency of environmental behaviors in six domains: transportation, food, energy and water saving, waste management, and sustainable purchase. Behaviors related to transportation (*M* = 3.34, *SD* = 1.35), food (*M* = 3.33, *SD* = 1.28), and sustainable purchases (*M* = 3.51, *SD* = 0.96) were reported to occur less frequently than behaviors related to energy (*M* = 4.25, SD = 0.98) and water saving (*M* = 4.45, *SD* = 0.88), and waste management (*M* = 4.20, *SD* = 0.96). To describe the relationship between these behaviors, we performed an exploratory factor analysis. We used principal axis factor analysis to extract factors for its relative tolerance of nonnormality and a Promax rotation since the factors are assumed to be correlated [85]. A scree plot indicated that five factors should be retained [86]. Items were assigned to the factors where they had the strongest loadings. Items with loadings below 0.4 were removed, which led to the removal of the following items: 3, 6, 8, 9, 19, and 20. After removal of these items, no loadings were salient on more than one factor.

**Fig 3:**
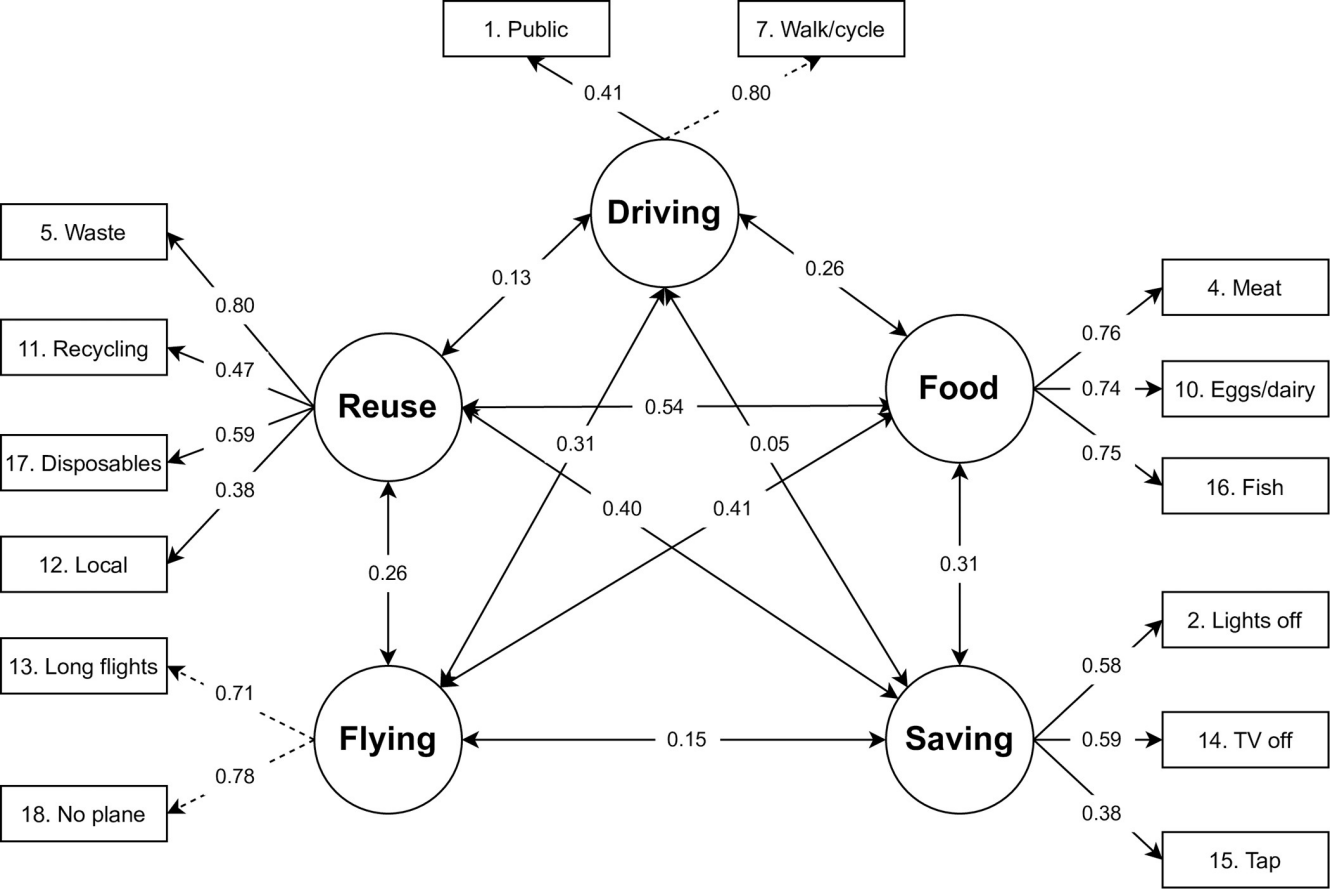
Pro-environmental behavior model. *Note*. Loadings, residual variances, and factor correlations are given as standardized parameters. Dotted lines indicate loadings that were constrained to be equal to allow for local factor identifiability.

Fig 3 presents the five-factor solution obtained. Factor 1 was named *Reuse* since items loading on it were related to sustainable waste management and investment in local products. Factor 2 was called *Driving* because items loading on this factor refer to reducing driving habits. Factor 3 was labeled *Flying* because items loaded on it mentioned reducing flying or choosing other transport instead of flying. Factor 4 was called *Saving* due to the items loading on it being related to actions that save water or electricity. Finally, Factor 5 was named *Food* because it captured items about changes in food choices. We ran a CFA with robust estimators on this solution. Model fit was acceptable, χ2 (70) = 141.201, CFI = 0.976, RMSEA = 0.033 [0.025,0.041], SRMR = 0.038. In general, factors were moderately correlated, except for the relation between Driving and Saving. Reliability results for our final factors were as follows: Driving (*r* = 0.38); Flying (*r* = 0.53); Food (α = .83, ωt = .83, GLB = .83); Saving (α = .63, ωt = .65, GLB = .65); Reuse (α = .71, ωt = .75, GLB = .72). Note that for factors with only two items, the reliability estimates represent the correlation between items.

### Psychological barriers

**Fig 4:**
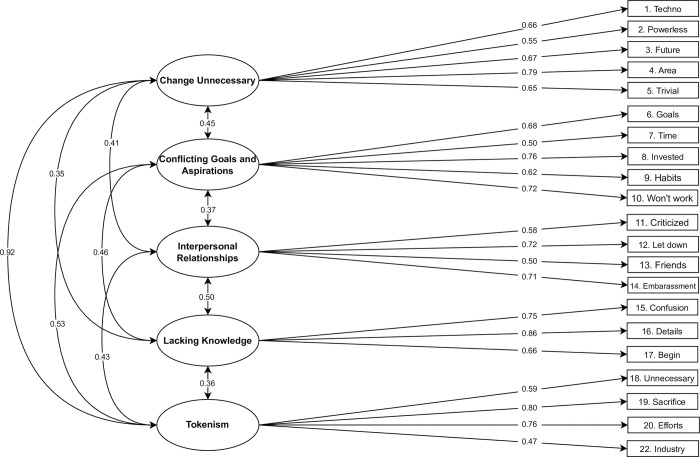
Psychological barriers model. *Note*. Loadings, residual variances, and factor correlations are given as standardized parameters.

We tested if we could replicate in our sample the five-factor structure found in Lacroix et al. [19]. The model with all 24 items included had a poor fit, χ2 (242) = 1223.564, CFI = 0.889, RMSEA = 0.066 [0.062,0.070], SRMR = 0.082. The analysis of the loadings and factor reliabilities indicated that the two additional items that were not part of the final DIPB scale (items 23 and 24) loaded poorly (<0.3) on their respective factors (standardized loading of 0.099 and 0.221, respectively). Removal of these items improved the reliability of the Tokenism (α = 0.82) and Lacking Knowledge (α = 0.85) latent factors, and produced a good fitting model, χ2 (199) = 493.105, CFI = 0.963, RMSEA = 0.040 [0.035,0.044], SRMR = 0.066. Yet, there was still an item (item 21) that showed a standardized loading of 0.265 which is below the criterion of 0.3. Since we are still left with four items in the tokenism factor, we decided to remove this low loading item, and its removal further improved the Tokenism factor’s reliability (α = 0.85). All other loadings were above 0.47. The final model with the removal of items 21, 23 and 24 had an acceptable fit: χ2 (179) = 464.670, CFI = 0.963, RMSEA = 0.041 [0.037, 0.046], SRMR = 0.069. Fig 4 displays the psychological barriers model with standardized loadings. As for the factor covariances, no covariance was negative, and the strongest relationship was found between *Change Unnecessary* and *Tokenism* (.92) and the lowest between *Change Unnecessary* and *Lacking Knowledge* (.35). Reliability results for our final factors were as follows: Change Unnecessary (α = .91, ωt = .93, GLB = .93); Conflicting Goals and Aspirations (α = .84, ωt = .88, GLB = .88); Interpersonal Relationships (α = .88, ωt = .92, GLB = .88); Lacking Knowledge (α = .85, ωt = .87, GLB = .86); Tokenism (α = .85, ωt = .68, GLB = .87).

**Fig 5:**
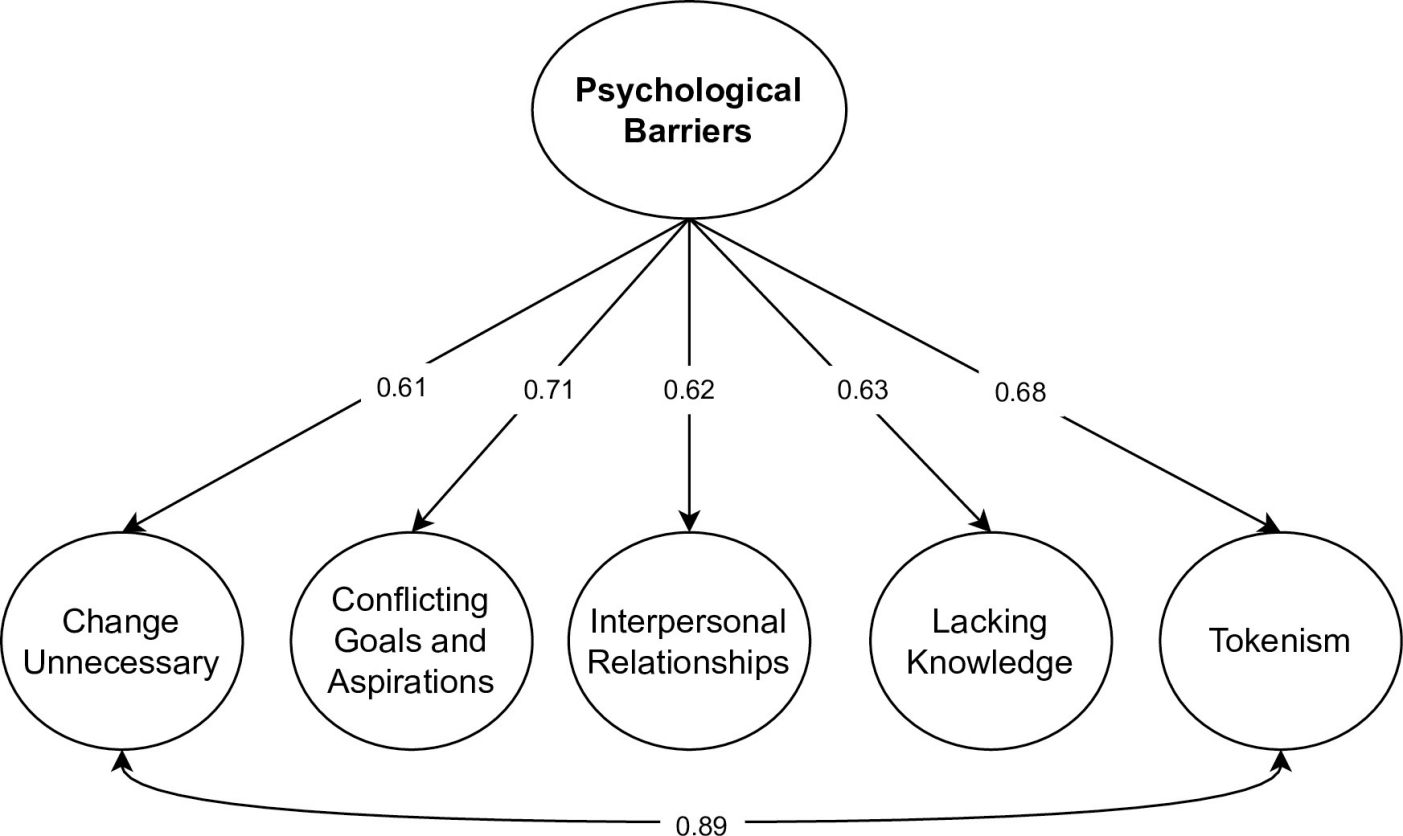
Second-order factor model for the psychological barriers scale. *Note*. Loadings are given as standardized parameters.

We also tested a second-order factor model where each of the five barriers loaded on a general psychological barrier factor. The model slightly misfitted the data, as indicated by the larger SRMR index (> 0.08), χ2(184) = 659.171, CFI = 0.928, RMSEA = 0.053 [0.067, 0.77], SRMR = 0.085. The modification indices indicated that model fit could be vastly improved if we considered the covariance between the Change Unnecessary and the Tokenism factors. The high correlation values between these two factors suggest a strong association. The DIPB scale measurement of Tokenism implies that current efforts are sufficient, and change is not necessary, which overlaps with the content of the Change Unnecessary factor and may explain some specific variance related to these factors. By including the covariance between Change Unnecessary and Tokenism, model fit improved, χ2(183) = 516.954, CFI = 0.956, RMSEA = 0.044 [0.040, 0.49], SRMR = 0.072. Fig 5 illustrates the second-factor model for the psychological barriers.

### Moderation analysis

Finally, our last aim was to assess if the psychological barriers moderated the influence of attitudes on the frequency of pro-environmental behaviors, thereby partly explaining the attitude-behavior gap. We performed a latent moderation analysis using a factor score procedure [87]. Although this approach neglects measurement error, given our sample size (*N* = 937) estimation error for the correlations in our sample should be low [88]. Simulations also show that this method performs relatively well compared to other methods even under suboptimal conditions, such as with lower reliabilities, null to medium correlations between the predictor and the moderator, and unequal factor loadings [87].

The latent moderation analysis involved three steps. First, we independently estimated the individual latent scores for each pro-environmental behavior factor separately using DWLS estimation. The models were saturated, and hence the fit was perfect. These latent scores served as our latent dependent variable in the moderation analysis. Second, we estimated individual factor scores for our latent predictors, namely Attitudes and Psychological Barriers, in a single model that allowed them to correlate. When estimating the model of Attitudes and Psychological Barriers together, the model did not converge if we included the correlation between the Change Unnecessary and Tokenism factors. Since the model without this correlation only showed a small misfit on its own, we proceeded with the estimation of the model without it. Model fit was acceptable, χ2(808) = 2456.02, CFI = 0.920, RMSEA = 0.047 [0.045, 0.49], SRMR = 0.078. The correlation estimated between attitudes and psychological barriers was *r* = – 0.46.

We entered the factor scores in a linear regression model of the following form:

Y(Behavior)=X(Concern)+Z(Barriers)+XZ

Table 4 contains the statistics for each regression model. The confidence intervals of 95% (CI) for each of the coefficients was obtained via bootstrapping with 1000 replications. *F*-statistic and adjusted R^2^ for each of the regression models was as follows, Reuse: *F*(3,933) = 48.95, *p* < .001, R^2^ = 0.13; Food: *F*(3,933) = 42.71, *p* < .001, R^2^ = 0.12; Saving: *F*(3,933) = 14.62, *p* < .001, R^2^ = 0.04; Flying: *F*(3,933) = 24.58, *p* < .001, R^2^ = 0.07; and Driving: *F*(3,933) = 4.62, *p* = .003, R^2^ = 0.01. Attitudes were a significant positive predictor of the frequency of all environmental behaviors except for Driving. Psychological Barriers were found to be a significant moderator for the relationship between Attitudes and the frequency of behaviors related to Reuse, Food, and Saving. Psychological Barriers did not significantly moderate the relationship between attitudes and Flying and Driving.

**Table 4:** Regression model results for each pro-environmental behavior.

Effect	Estimate	CI	*SE*	t	*p*
**Reuse**					
Intercept	- .022	[-.067; .025]	.027	- 0.805	.421
Attitudes	.369	[.277; 0.460]	.041	8.894	< .001*
Psychological Barriers	- .047	[-.116; .032]	.035	- 1.337	.182
Moderation	- .040	[-.071; -.008]	.016	-2.582	.01*
**Food**					
Intercept	- .025	[-.073; .024]	.029	-0.880	.379
Attitudes	.318	[.237; .396]	.044	7.288	< .001*
Psychological Barriers	-.120	[-.175;—.056]	.037	-3.229	.001*
Moderation	-.046	[-.070; -.018]	.016	-2.822	.005*
**Saving**					
Intercept	-.021	[-.067; .024]	.025	-0.815	.415
Attitudes	.226	[.145; .306]	.038	5.861	< .001*
Psychological Barriers	.006	[-.048; .059]	.033	0.181	.856
Moderation	-.038	[-.077;—.001]	.015	-2.614	.009*
**Flying**					
Intercept	-.003	[-.048; .043]	.027	-0.125	.901
Attitudes	.271	[.196; .346]	.041	6.616	< .001*
Psychological Barriers	.050	[-.001; .106]	.035	1.423	.155
Moderation	-.006	[-.031; .022]	.015	-.400	.689
**Driving**					
Intercept	.004	[-.044; .050]	.028	0.164	.870
Attitudes	.060	[-.006; .132]	.042	1.422	.155
Psychological Barriers	-.038	[-.091; .018]	.036	-1.037	.300
Moderation	.008	[-.018; .033]	.016	0.525	.600

**Fig 6:**
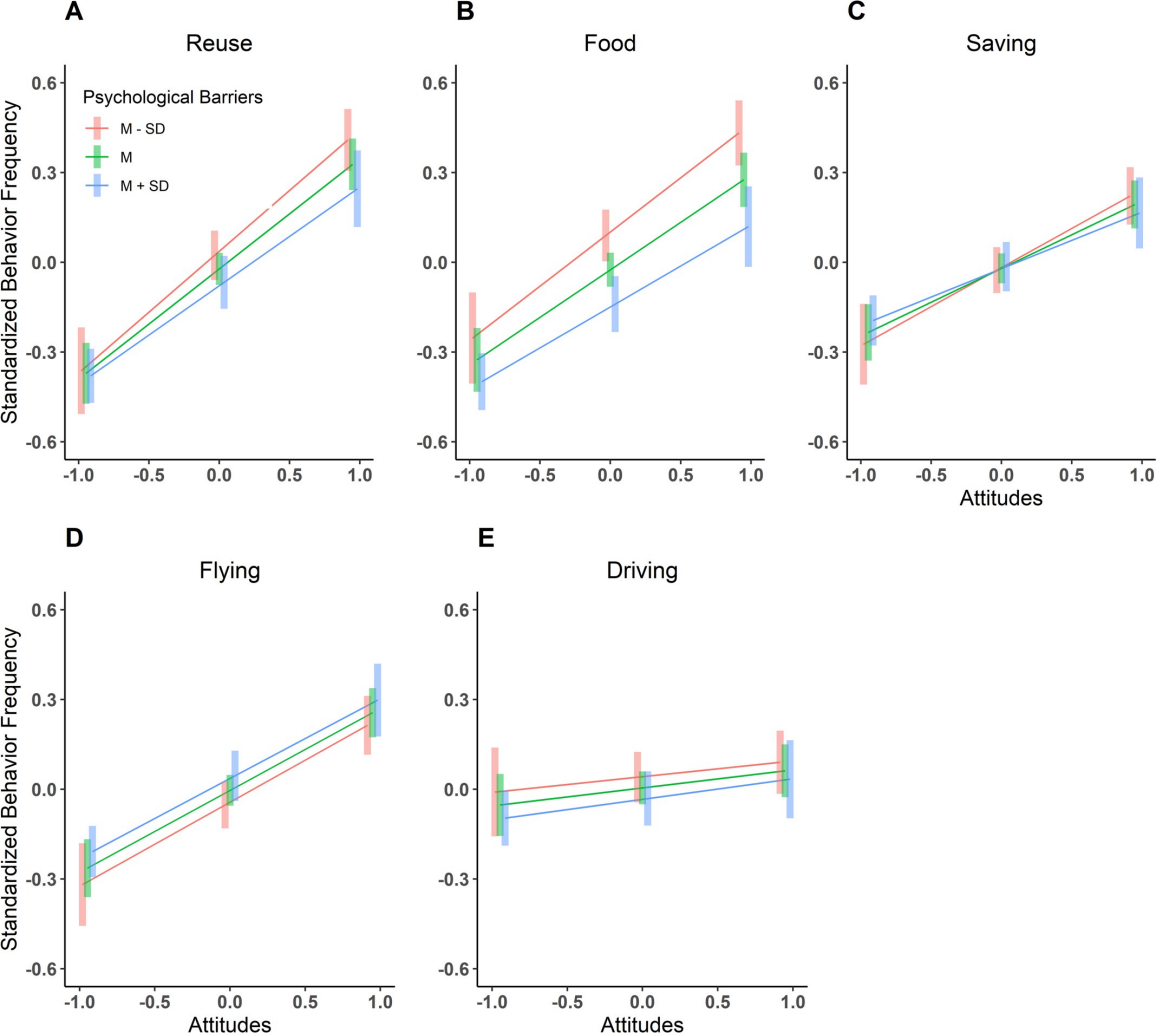
Linear relationships between attitudes and pro-environmental behavior for different levels of psychological barriers. *Note*. The vertical lines represent the 95% confidence intervals for the standardized behavior frequency across different levels of psychological barriers.

We graphicly illustrated the moderation effect of Psychological Barriers for each Pro-Environmental Behavior in Fig 6. Linear predictions between Attitudes (independent variable) and Pro-Environmental Behavior (dependent variable) were computed for those with low, average, and high levels of Psychological Barriers (i.e., one SD below the mean, the mean, and one SD above the mean, respectively). For Reuse and Food, the effect of psychological barriers mattered most at high levels of environmental attitudes. For Saving, the pattern with the effects of psychological barriers reversed for high and low levels of environmental attitudes.

## Discussion

In this study, we evaluated whether psychological barriers could partly explain the gap between environmental attitudes and engagement in different domains of environmental action. We assessed environmental attitudes as the latent factor representing the covariation of environmental concerns and climate change beliefs. We used this model to assess if favorable environmental attitudes relate to a higher frequency of climate action. Finally, we examined if psychological barriers, as assessed by the DIPB scale, moderated the relation between attitudes and behavior frequency in each domain of action. Next, we will first discuss our findings regarding the relationship between attitudes and behavior, followed by how psychological barriers may moderate it.

### Environmental attitudes and behaviors

Our sample of Portuguese individuals was highly concerned about climate change and believed in the human impact on this phenomenon. Climate change beliefs were very high on all levels, and environmental concerns were higher in the biospheric and altruistic domains than the egoistic one. Previous studies indicated that biospheric and altruistic values tend to be the most correlated with the intention to act [32, 40], yet all concerns are usually correlated to each other [35, 41–43]. Accordingly, we were able to extract a single factor reflecting pro-environmental attitudes from the measures of climate change beliefs and environmental concerns.

The frequency of pro-environmental behaviors showed some variability. Behaviors with lower carbon impact, such as reducing water use, conserving energy, and recycling or using less plastic, were done more frequently than reducing car use, airplane traveling, and eating less meat or animal products. These results align with the idea that high carbon impact behaviors require more investment to change [10, 48].

Positive environmental attitudes predicted a higher frequency of environmental behavior across all our tested domains except for behaviors related to driving less. However, it is worth pointing out that the association between attitudes and self-reported frequency was not very high (estimates ranged between .025 and .381), which is consistent with previous reports of a modest association between environmental attitudes and behavior [12].

### Psychological barriers

The DIPB scale was created to evaluate psychological barriers across different environmental behavior domains and was originally developed and tested in a Canadian sample [19]. We were able to replicate the original 5-factor structure in our Portuguese sample with some minor model adaptations. This pattern is similar to the one obtained in a Colombian sample [65]. The short format of the DIPB scale and the generality of the 5-factor solution extracted from it suggests that the scale is useful to, briefly and efficiently, measure psychological barriers to pro-environmental action in diverse cultural contexts.

In our sample, change unnecessary and interpersonal relationships were overall the lowest-rated barriers, while tokenism, lacking knowledge, and conflicting goals were the strongest barriers. It is worth noting that the items concerning the perceived government’s duty in facilitating action (items 23 and 24) were removed due to low model adequacy, but these were the highest-rated barriers to pro-environmental behavior in our sample. We believe the DIPB scale did not capture this phenomenon well, but the dismissal of personal responsibility might be a promising psychological barrier to be further explored in the future. An Australian study [89] showed that attributing greater responsibility to the government for environmental protection was related to more negative environmental intentions and behavior than attributing responsibility to the community. Although the measurement of this barrier through items 23 and 24 did not work well within the context of the DIPB scale, it does not mean that this barrier is not a relevant determinant of environmental action. Future studies should consider how to measure this psychological barrier effectively.

In our study, the psychological barriers moderated the effect of attitudes on behavior domains like waste management (reuse factor), eating less meat (food factor), and conservation behaviors (saving factor). Psychological barriers had a stronger influence on individuals with a higher environmental positive attitude, indicating that they may help explain the attitude-behavior gap that prevents concerned individuals from effective action. This relation was clear for the reuse and food factors but less so for the saving factor. Critically, this moderation was not significant in the transportation domains (car usage and plane traveling). The moderation effects observed here align with our predictions that psychological barriers may partly explain the attitude-behavior gap in some behaviors but not others. The transportation and food domains are considered of high difficulty to change [19]. Nevertheless, against our expectations, we only observed a moderation effect for food choices, but not driving or flying. This may suggest that although both behaviors are of high difficulty and have a high impact on the carbon footprint, they cannot be explained in the same way.

On the one hand, in our analysis, food habits were explained by attitudes with psychological barriers moderating that relationship. In the literature, meat-eating behavior interventions appear often associated with values and attitudes [90]. For example, self-transcending values effectively increase negative attitudes toward meat consumption [91], and attitudes about animals are important in choosing vegetarianism [92]. Furthermore, lack of knowledge (which is one of the assessed psychological barriers in our study) usually acts as a barrier to decreasing meat intake; and interventions focused on increasing awareness coupled with knowledge on how to replace meat with less impactful food choices increase positive attitudes and intentions towards meat reduction [90, 93].

On the other hand, our analysis indicates that transportation behaviors (i.e., flying and driving) were less explained by attitudes, and no moderation effect was observed. The willingness to reduce flying was positively associated with attitudes, yet this behavior was not moderated by psychological barriers. Unfortunately, we recorded no information about the flying habits of our sample. It has been observed that habitual plane traveling is associated with a higher resistance to reducing flying in the future compared to those who air travel only occasionally [94] or prefer other means of transportation [95]. Flight reduction is also associated with how accessible and efficient the alternatives are (e.g., train) [94]. Hence it is possible that our results could be explained by the air travel habits of our sample. Future studies should control the frequency of air travel to assess if psychological barriers play different roles depending on how much people use this mode of transportation. Driving was the only domain not predicted by climate change attitudes. This result might point to other stronger motives behind driving habits that are not covered by our scales. For example, Innocenti et al. [96] found that people are biased against public transportation, preferring to travel by car even when the costs of this option are higher. A possible explanation is that cars and driving symbolize freedom, independence, status, and a pleasurable activity [97]. Other factors, such as travel time, which is longer for public transportation, are also crucial when choosing a traveling mode [98]. As such, people with complex activity patterns (e.g., taking children to school before going to work) might prefer to avoid the rigid and unreliable public transportation system, preferring to travel by personal transportation, which can be either bike or car. Note that bike traveling is also limited by the existence of safe and adequate bike lanes [99]. As for flying, we have not assessed the preferred transportation modes of our sample. Hence, we could not test whether psychological barriers vary depending on the driving habits of our participants. Future studies should consider measuring this variable.

A meta-analysis on behavioral interventions to promote household action on climate change pointed out that interventions on reducing meat eating tend to be more effective than reducing private car usage [100]. Interventions on meat reduction usually center around removing external barriers/facilitating mitigation behavior, while transportation interventions revolve mostly around information-based interventions, increasing the appeal of not using cars. These studies indicate that while food mitigation behaviors can be mostly linked with the variables assessed by our study, driving and flying habits might be associated with social status, previous behaviors, and practicality over alternative methods (i.e., public transport, walking, riding a bike), which were not evaluated here and may well explain why we could not effectively capture the motives behind driving and flying habits.

### Limitations and future directions

Collecting data via a survey has its limitations. Self-reported measures of pro-environmental behavior might not entirely reflect authentic environmental action as it may depend on the participant’s interpretation of the question and recollection of their activity [101] or be influenced by social desirability [102]. It remains, therefore, a challenge to objectively monitor the frequency of pro-environmental action. Nonetheless, we forward some promising alternative methods. Laboratory observations through experimental tasks do not rely as much on interpretation and offer experimenters higher degrees of control [101]. Laboratory tasks usually rely on assessing participants’ decisions in different manipulated scenarios. For example, the Pro-Environmental Behavior Task [103] requires participants to make several trips. On each trip, they can choose between an environmentally friendly or unfriendly mode of transportation with manipulated waiting periods. Another way to curb recollection and interpretation restrictions is through momentary ecological assessment using mobile devices to register behaviors while they are happening [104].

There may be some limitations of generalizability related to our sample demographics. We collected data in Portugal from relatively young, mostly female, urban, and digitally active individuals (since this study was advertised via email and social media). Our results therefore may not generalize to individuals with different demographic characteristics. We note, however, that we replicated with our sample the main results observed in other studies with North-American and Latin-American samples [19, 65]; hence our findings are likely to generalize across cultures.

This study showed that the DIPB scale has some degree of explanatory power toward the existence of a gap between environmental attitudes and (self-reported) ecological behavior. However, future studies could integrate these barriers into a broader model framework, such as the comprehensive action determination model [26]. The effects of habits and perceived behavioral control were shown to have a direct relationship with pro-environmental behavior [12], and their inclusion in this model framework might add explanatory power to the attitude-behavior gap.

Furthermore, we lacked a measure of structural barriers. Future studies should consider including a measure of structural and external barriers to better separate the influence of psychological barriers from other factors that may prevent people from taking more action. A lack of accessibility to infrastructures might be increasing behavior costs. For example, enhanced accessibility to recycling facilities encourages people to recycle [105]. Low availability of public transportation can be responsible for increased car use in rural areas [61], while product prices impact what people eat [57]. Hence, the accessibility of some structures or products may help explain the lack of action from concerned individuals beyond intrinsic barriers.

A final note regarding the measurement of psychological barriers falls upon the necessity to develop more robust ways to study people’s tendency to place responsibility for action on the government and industry, which was not well assessed by the DIPB scale but had a strong presence in our sample.

## Conclusions

The present study enhanced our understanding of the relationship between attitudes, psychological barriers, and pro-climate behaviors. We demonstrated that not all types of behaviors are equally influenced by attitudes and the perception of psychological barriers. The DIPB scale could, to some degree, be a helpful tool to explain the attitude-behavior gap. This knowledge will help to plan more focused and efficient interventions which focus on breaking psychological barriers. Behaviors related to food choices, waste management, or water and energy saving can benefit from an intervention related to increasing attitudes while not ignoring the potential effects of psychological barriers. Finally, driving seems to be more resistant to change, and merely stimulating positive environmental attitudes or dismantling psychological barriers may not be enough.
